# Risk of Cardiovascular Events in Adults Aged 40 to 79 Years with Diagnosed Hypertension, High Cholesterol, and/or Diabetes but Not on Medications: Findings from Nationwide Cross-Sectional Studies

**DOI:** 10.3390/jcdd11090268

**Published:** 2024-08-29

**Authors:** Shuting Wang, Yanji Qu, Jiayue Zhang, Xue Xue, Zuyao Yang

**Affiliations:** 1 JC School of Public Health and Primary Care, The Chinese University of Hong Kong, Hong Kong 999077, China; wangst@link.cuhk.edu.hk (S.W.); xuexue@cuhk.edu.hk (X.X.); 2 Global Health Research Center, Guangdong Cardiovascular Institute, Guangdong Provincial People’s Hospital, Guangzhou 510000, China; quyanji@gdph.org.cn; 3 Faculty of Medicine, Macau University of Science and Technology, Macao 999078, China; 3230004421@student.must.edu.mo; 4 Department of Nephrology, Affiliated Hospital of Hubei University of Chinese Medicine, Hubei Provincial Hospital of Traditional Chinese Medicine, Wuhan 430061, China

**Keywords:** hypertension, high cholesterol, diabetes, cardiovascular risk, risk management

## Abstract

Many people with diagnosed hypertension, high cholesterol, and/or diabetes are not receiving drug treatment, partly because they perceive their cardiovascular disease (CVD) risk as low. This study aimed to quantify the risk for future CVD events, either first or recurrent, in people with diagnosed hypertension, high cholesterol, and/or diabetes but not on medications for any of these conditions. Participants aged 40–79 years who had been diagnosed with hypertension, high cholesterol, and/or diabetes but were not on medications were identified from National Health and Nutrition Examination Surveys cycles 1999 to 2018. Among them, those with known CVD and those without known CVD but with complete data for estimating their 10-year CVD risk were included in this study. The participants were classified as (1) “high-risk” if they had known CVD or a 10-year predicted CVD risk ≥ 7.5% or (2) “low-risk” if they had a 10-year predicted CVD risk < 7.5%. Of the 5187 participants included, 2201 had known major CVD (n = 490, 9.45%) or a 10-year predicted CVD risk ≥ 7.5% (n = 1711, 32.99%), corresponding to a weighted proportion of 34.83% (95% CI: 33.15 to 36.51%) in the US general population. The proportions of high-risk participants were much higher in the elderly (65.50% for 60–69 years and 97.86% for 70–79 years), males (45.13%), and non-Hispanic Blacks (42.15%) than in others (all *p* < 0.001). These patterns were consistent across survey cycles during 1999–2018. Additional analyses that classified the participants into groups above or below the treatment threshold (rather than high- or low-risk groups) according to current guidelines yielded similar results. A comparison of the 2201 untreated high-risk participants with other participants who had been diagnosed with hypertension, high cholesterol, and/or diabetes and were on medications for these conditions showed that “lower BMI”, “smaller waist circumference”, and a “non-diabetic” status, among others, were associated with a higher likelihood of “not taking medications”. In conclusion, approximately one-third of the US adults aged 40 to 79 years with diagnosed hypertension, high cholesterol, and/or diabetes but not on medications had known CVD or a 10-year predicted CVD risk ≥ 7.5%, and this proportion was little changed over the past two decades. Interventions targeted at the subgroups with particular characteristics identified in this study may help improve the management of CVD and its risk factors.

## 1. Introduction

Hypertension, high cholesterol, and diabetes are major modifiable risk factors for cardiovascular disease (CVD). These three conditions constitute a heavy burden on global health [1] and often coexist within one individual due to shared lifestyle and pathophysiological factors [2,3]. Drug treatments of these conditions can reduce CVD events in both primary and secondary prevention settings [4,5,6,7] and are usually recommended for patients with known CVD or those with a high 10-year predicted risk of developing CVD. For instance, the 2019 American College of Cardiology/American Heart Association (ACC/AHA) guideline on the primary prevention of CVD recommends statin therapy to those who have clinical atherosclerotic CVD or a 10-year CVD risk ≥ 7.5%, estimated by the Pooled Cohort Equations (PCE) [8], which is a well-calibrated prediction tool to estimate the 10-year risk of developing a first atherosclerotic CVD event in the US general population [9].

However, the treatment and control rates of these three conditions are not satisfactory in the real world, even among patients with CVD. For example, in the US, only about 30% of the patients with hypertension or diabetes had these conditions well controlled [10], and only 21% of patients with diagnosed diabetes achieved the control goals of hemoglobin A1c, blood pressure (BP), and low-density lipoprotein cholesterol levels [11]. Among others, one reason for the suboptimal control of these conditions is that some patients are reluctant to receive drug therapy because they are not aware of their CVD risk or they perceive their risk of future CVD events as low [8,12]. Thus, it would be of interest to assess the risk for CVD events—either first or recurrent—in these patients and identify the high-risk ones who are in need of drug treatment. High-risk patients can be those without known CVD (who are susceptible to a first attack) or those with established CVD (who are susceptible to a recurrent attack). This study included US adults aged 40 to 79 years with hypertension, high cholesterol, and/or diabetes but not on medications, with or without known CVD, to estimate their risk for future CVD events. The characteristics of these untreated high-risk patients were compared with those who were taking medications to identify the factors related to medication-taking behaviors, which can inform the development of targeted interventions.

## 2. Materials and Methods

### 2.1. Data Sources and Study Population

**Figure 1:**
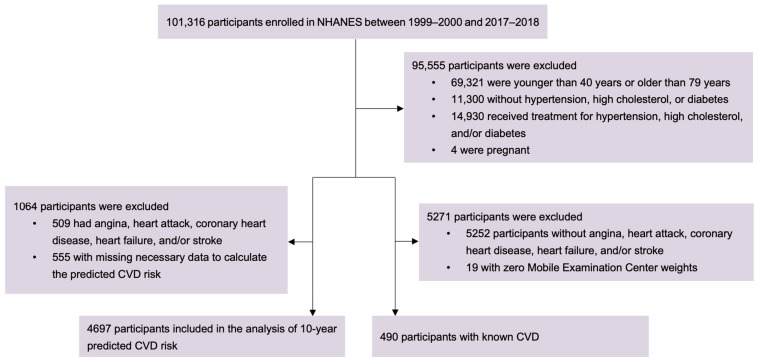
Flowchart of the inclusion and exclusion of participants.

The data used in this study were collected in 10 National Health and Nutrition Examination Surveys (NHANES) cycles between 1999–2000 and 2017–2018. The NHANES uses a complex, stratified, multistage probability cluster sampling design to select a representative sample of noninstitutionalized citizens living in the US. The detailed study design and data of the NHANES are publicly available at https://www.cdc.gov/nchs/nhanes/ (accessed on 18 March 2024). The target population in the present study is participants who had been diagnosed with at least one case of hypertension, high cholesterol, and diabetes (who were potential recipients of drug treatments for these conditions) but were not actually on medications, regardless of whether they had known CVD. Participants enrolled in the 10 NHANES cycles from 1999 to 2018 who (1) were aged between 40 and 79 years (to be consistent with the age range to which PCE is applicable [9]); (2) had been told by a doctor to have at least one of the following conditions: hypertension, high cholesterol level, and diabetes; (3) were not receiving drug treatment for any of these conditions; and (4) were not pregnant were eligible for this study. In total, 5187 participants were included. The flowchart of the participant selection is presented in Figure 1. Among them, 4697 participants who were free of major CVD (any of the following: angina, heart attack, coronary heart disease, heart failure, and stroke) were at risk of a first CVD attack, while the other 490 participants who had known major CVD were at risk of recurrent CVD attacks.

### 2.2. Variables and Prediction of 10-Year CVD Risk

For each participant who was free of major CVD, the 10-year predicted CVD risk was estimated using the sex- and race-specific PCE models, as recommended by the ACC/AHA guideline [9]. The variables used in PCE models included age, sex, race (i.e., non-Hispanic White, non-Hispanic Black, or others), diabetes status, antihypertensive drug treatment, current smoking status, total cholesterol (TC, mg/dL), high-density lipoprotein cholesterol (HDL, mg/dL), and systolic BP (mm Hg).

Age, sex, race, histories of doctor-diagnosed hypertension, high cholesterol level, and/or diabetes, current use of medications for these conditions, and histories of doctor-diagnosed major CVD (defined as the presence of any of the following: angina, heart attack, coronary heart disease, heart failure, and stroke) were self-reported by participants at the time of the survey. The participants who were not known to have hypertension, high cholesterol, and/or diabetes until they were assessed during the NHANES were not included in this study, because this study aimed to identify high-risk patients who knew that they had at least one of these conditions (as indicated by their self-reports) but somehow did not take medications, which could provide implications for improving the treatment rate in such population. For those who did not know that they had one or more of the three conditions before, the reason that they were not on medications could simply be a lack of detection and awareness of the disease(s), which was not the focus of this study. A participant was classified as a current smoker if he/she self-reported that had smoked at least 100 cigarettes in life and was currently smoking cigarettes at the time of the survey. The TC and HDL were measured enzymatically. The BP was measured by trained physicians using a mercury sphygmomanometer with an appropriately sized cuff after resting quietly in a seated position for 5 min at each cycle of physical examination. The mean systolic BP was calculated as the average of up to 4 readings obtained in a single physical examination [10,13,14].

Other covariates used to describe the characteristics of participants in different CVD risk groups and analyzed as potential factors contributing to higher CVD risk included marital status (married vs. others), education (college degree and above vs. others), body mass index (BMI) (categorized as normal if <25.0 kg/m^2^, overweight if 25.0–29.9 kg/m^2^, and obese if ≥30.0 kg/m^2^), waist circumference (cm), physical activity (sedentary, moderate, and vigorous), current drinking status (whether drinking alcohol during the past 12 months: yes vs. no). The data on marital status, education, physical activity, and current drinking status were self-reported by the participant during the interview. The BMI and waist circumference were measured by trained health technicians.

### 2.3. Statistical Analysis

The outcomes of interest in this study were the proportions of participants in different CVD risk groups. According to the ACC/AHA guideline on the primary prevention of CVD, 7.5% and 10% were adopted as the statin treatment threshold and antihypertensive treatment threshold, respectively [8]. Therefore, for those without known major CVD, the 10-year predicted CVD risk was categorized into 5 levels, i.e., <5%, 5 to 7.4%, 7.5 to 9.9%, 10 to 19.9%, and ≥20%. Those who had a 10-year predicted CVD risk of <7.5% were collectively referred to as low-risk participants, while those who had a 10-year predicted CVD risk of ≥7.5% and those with diagnosed major CVD by the time of the survey were collectively referred to as high-risk participants in this study. To account for the potential oversampling of certain populations and nonresponse in the complex, multistage NHANES, the mobile examination center sample weights were incorporated when calculating the proportion of each CVD risk group. The Chi-squared test was used to assess the differences between subgroups in the distribution of CVD risk.

In clinical practice, the decision on whether to recommend to a person with hypertension, high cholesterol level, and/or diabetes is not made solely on the basis of the person’s CVD risk but would also take into account other factors, such as comorbidities [8,15]. In view of this, we performed additional analysis to estimate the proportions of participants who were “above treatment threshold” and “below treatment threshold”, respectively. Those who (1) had known major CVD [15,16]; (2) had concurrent high cholesterol and diabetes, or concurrent hypertension and diabetes [8]; (3) had no high cholesterol but had hypertension and a 10-year predicted risk ≥ 10% [8]; or (4) did not fulfill criteria 1 to 3 but had a 10-year predicted risk ≥ 7.5% were considered “above treatment threshold” [8], while the others were considered “below treatment threshold”.

In another additional analysis, we additionally included the NHANES participants who had been diagnosed with hypertension, high cholesterol, and/or diabetes and were taking medications and compared them with those who did not take medications but were at high risk of future CVD as identified in the main analysis. The aim was to identify the characteristics associated with not taking medications in high-risk patients. The weighted multivariable logistic regression accounting for the complex sampling was adopted for this comparison, and the weighted odds ratio (OR) with 95% confidence interval (CI) was estimated for each covariate included in the model. In case of missing data on covariates, a complete case analysis was adopted in the regression. We also performed sensitivity analysis with missing data imputed to examine the robustness of the results.

All statistical analyses were performed using SAS version 9.4 (SAS Institute Inc., Cary, NC, USA), except for the missing data multiple imputation, which was performed using the “mice” package in R software version 4.3.1. A 2-sided *p* value < 0.05 was considered statistically significant. Ethical approval was exempted as this study used free, public, and de-identified datasets from the NHANES.

## 3. Results

The characteristics of the 5187 participants included in this study are presented in Table 1. The mean age was 59.94 years. Approximately half of them were male, non-Hispanic White, married, and had a college degree or above. Notably, 41.79%, 76.08%, and 6.84% had hypertension, high cholesterol level, and diabetes, respectively, and 17.81% had at least two of these conditions. Of the participants, 45.00%, 12.57%, 8.35%, 16.04%, and 8.60% had a 10-year predicted CVD risk of <5%, 5 to 7.4%, 7.5 to 9.9%, 10 to 19.9%, and ≥20%, respectively, and 9.45% had known major CVD (Table 1). The mean risk was 3.1% and 16.5% in those with a 10-year predicted CVD risk of <7.5% and ≥7.5%, respectively. In the participants with only one of the three conditions, the mean risk was 7.4%, and 31.36% of them had a 10-year predicted risk ≥7.5%. In those with two or three conditions, the mean risk was 10.7%, and 40.48% of them had a 10-year predicted risk ≥7.5%.

The weighted proportion was 65.17% (95% confidence interval [CI]: 63.49 to 66.85%) for low-risk participants (10-year predicted CVD risk < 7.5%) and 34.83% (95% CI: 33.15 to 36.51%) for high-risk ones (10-year predicted CVD risk ≥ 7.5% or with known major CVD), corresponding to 15,188,288 and 8,117,071 adults in the US general population, respectively. The weighted proportion for participants with 10-year CVD risk ≥10% or known major CVD was 26.49% (95% CI: 25.01 to 27.98%), corresponding to 6,174,119 adults in the US general population.

**Figure 2:**
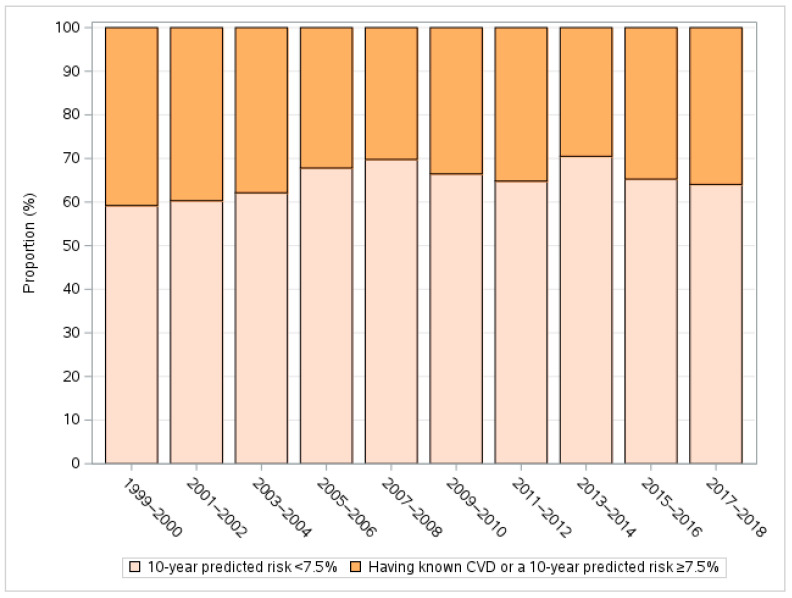
Distribution of CVD risk in untreated patients with diagnosed hypertension, high cholesterol, and/or diabetes during 1999 and 2018.

**Figure 3:**
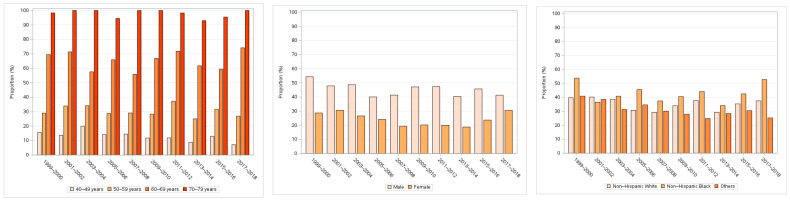
The proportion of participants who had known CVD or a 10-year predicted risk ≥7.5% by age group, gender, and race between 1999 and 2018.

The proportion of high-risk participants was largely stable over the 20 years covered by this study (Figure 2) but increased with age groups from 13.03% (95% CI: 11.07 to 14.98%) in the group aged 40–49 years to 97.86% (96.16 to 99.56%) in the group aged 70–79 years (*p* < 0.001) and was significantly higher in males (45.13% vs. 24.29% in female, *p* < 0.001) and non-Hispanic Blacks (42.15% vs. 35.20% in non-Hispanic White and 30.32% in other races, *p* < 0.001) (Figure 3 and Table 2). The patterns of CVD risk distribution across subgroups defined by these factors were consistent over the study period (Figure 3).

Consistent with the main results, sensitivity analysis showed that 33.77% (95% CI: 32.15 to 35.39%) of the participants were above the treatment threshold recommended by current guidelines, and the proportion of participants above the treatment threshold also increased with age group and was significantly higher in males and non-Hispanic Blacks (all *p* < 0.001, Supplementary Table S1).

Additional analysis showed that, compared with those who took medications, high-risk patients who were not on medications were more likely to be older (weighted OR = 1.35 for ages 55 to 64 years; weighted OR = 1.84 for ages ≥ 65 years), male (weighted OR = 2.12), non-Black (weighted OR = 1.27 for non-Hispanic White; weighted OR = 1.47 for other ethnicities), unmarried (weighted OR = 1.50), current smokers (weighted OR = 2.62), have a lower BMI (weighted OR = 1.28 for overweight; weighted OR = 1.45 for normal weight), have smaller waist circumference (weighted OR = 1.24 for 100 to 109 cm, weighted OR = 1.50 for 90 to 99 cm; weighted OR = 1.50 for less than 90 cm), non-diabetic (weighted OR = 3.88), have a higher TC-to-HDL ratio (weighted OR = 3.74), and have a systolic BP ≥ 140 mmHg at the time of survey (but not necessarily had diagnosed hypertension) (weighted OR = 1.53) (Table 3). In the sensitivity analysis with missing data for covariates imputed, two ORs for waist circumference became statistically insignificant, but their direction and trend were unchanged, and all the other factors mentioned above remained statistically significantly associated with being high-risk and untreated (Supplementary Table S2).

## 4. Discussion

We found that approximately one-third of the patients with hypertension, high cholesterol, and/or diabetes who were not receiving drug treatment for any of these conditions had a 10-year predicted CVD risk ≥ 7.5% or known CVD, and this proportion was little changed over the past two decades. Assuming that this one-third population (mean CVD risk: 16.5%) received identical treatments for two of the three conditions as in the trial conducted by Yusuf et al. [17], where the relative risk reduction in CVD events was around 30%, the absolute risk reduction would be around 5% (=16.5% × 30%), corresponding to 405,853 (=8,117,071 × 5%) CVD events prevented in 10 years in the US. This number could be slightly smaller in reality, given the fact that most people had only one of the three conditions (Table 1). Regardless, this finding highlights the need for improvement in the management of CVD and their risk factors in the US. To achieve this, it is important to accurately assess individual CVD risk in primary prevention and improve the risk communication to increase the high-risk patient’s willingness to receive drug treatment in both primary and secondary prevention of CVD.

The larger proportions of high-risk participants in older age groups and males were anticipated, because they generally have a higher CVD risk [18,19,20]. However, it should be noted that using chronological age in the PCE may lead to nearly all men in their mid-to-late 60s and nearly all women in their 70s exceeding the 7.5% risk threshold, even if they have an optimal risk factor profile [21]. This may explain the particularly high percentage (97%) of high-risk participants in the age group of 70 to 79 years in this study. It has been suggested that a broader construct of age, such as “heart age” and “vascular age” [21], or a higher risk threshold in older patients [22], may help make wiser treatment decisions, as people age differently. The finding that non-Hispanic Blacks constituted the largest proportion (42%) of high-risk participants was consistent with previous studies [23,24] and could have been driven by social determinants of health such as low income and poor access to health care [24].

One reason that high-risk patients did not receive drug treatment is their lack of awareness of their own CVD risk. Previous studies showed that people often underestimated their CVD risk due to “optimistic bias” [12,25]. This is consistent with our findings from the comparison of the high-risk patients who did not take medications (i.e., the main analysis population) and those who took medications. Notably, patients who did not take medications had some “heart-healthy” characteristics such as lower BMI, smaller waist circumference, and normal glycemic levels (Table 3), while around one-third of them were, in fact, at a high risk of future CVD events. The high-risk patients may have mistakenly perceived their CVD risk to be low due to those preferable characteristics and consequently considered medications to be unnecessary. To address this issue, more efforts to improve risk communication should be targeted to this particular population in both primary and secondary prevention of CVD. For example, contextualizing the 10-year predicted risk in the form of risk relative to peers may improve risk understanding and the willingness to receive drug treatment [12]. Other barriers to drug treatment, such as fear of side effects, lack of understanding about benefits, and financial burdens, should also be addressed [25,26]. On the other hand, it is noteworthy that the majority of the participants in this study were at a relatively low risk of CVD. For these people, lifestyle modifications including nutrition, physical activity, weight loss, and smoking cessation may be more cost-effective in the primary prevention of CVD [8].

This study has several limitations. First, the use of medication was self-reported and thus subject to recall bias. However, previous studies have reported a good agreement between self-reported medication use and prescribing data, including the use of antihypertensive drugs, cholesterol-lowering medications, and insulin [27,28]. Second, the diagnosis of hypertension, high cholesterol, diabetes, and CVD was also self-reported. Previous studies showed that people tended to under-report the presence of CVD risk factors (including hypertension, high cholesterol, and diabetes) [29] and less serious CVD [30], which means that the CVD risk of our study population might have been underestimated. Third, the severity of the three conditions and the treatment side effects, such as muscle weakness and lack of sex drive associated with statins, are common reasons why patients choose not to take medications. However, as data on these factors were available for only a few participants (n = 28), and there was insufficient information to determine whether they were caused by the use of medications, we were not able to adjust for them in our multivariable analyses to investigate the factors associated with being at high risk but not receiving drug treatment. Fourth, the NHANES collected no data on the specific type and dosage of drugs, such as the use of statins. Thus, the factors associated with the use of a particular drug were not able to be explored in our study. Fifth, the study sample underwent several rounds of selection, and participants who did not fulfill the inclusion criteria or had missing data on certain variables were excluded. The effect of non-response bias may not have been fully accounted for, although appropriate sampling weights were used to minimize non-response bias in the analyses. Sixth, it was reported that the PCE may overestimate the risk in certain groups [9], but a recent study that assessed the performance of PCE using a sample of 30,000 adults in clinical practice settings found a very good calibration of the 10-year predicted risk around the threshold of 7.5% across different sex and race groups [31]. Thus, we believe that the potential overestimation of CVD risk by using PCE did not significantly affect the categorization of low- and high-risk groups in this study.

In conclusion, approximately one-third of the US adults aged 40 to 79 years who had been diagnosed with at least one of hypertension, high cholesterol, and diabetes before but not on medications had known CVD or a 10-year predicted CVD risk ≥7.5%, especially in older people, males, and non-Hispanic Blacks. This situation has not changed much over the past two decades. Characteristics associated with not taking medications in high-risk patients were identified. Interventions targeted at these characteristics are warranted to improve the management of CVD events and their risk factors.

## Figures and Tables

**Table 1:** The characteristics of the study population.

Baseline Characteristics	10-Year CVD Risk Predicted by Pooled Cohort Equation					People with Known Major CVD (n = 490, 9.45%)	Total (n = 5187, 100%)
	<5% (n = 2334, 45.00%)	5 to 7.4% (n = 652, 12.57%)	7.5 to 9.9% (n = 433, 8.35%)	10 to 19.9% (n = 832, 16.04%)	>20% (n = 446, 8.60%)		
Age, mean (SD)	48.39 (6.23)	54.39 (7.58)	57.15 (7.92)	61.93 (8.19)	69.29 (7.82)	59.99 (11.07)	54.94 (10.26)
Male, n (%)	825 (35.35)	378 (57.98)	263 (60.74)	595 (71.51)	319 (71.52)	273 (55.71)	2653 (51.15)
Race, n (%)							
Non-Hispanic White	1016 (43.53)	299 (45.86)	203 (46.88)	352 (42.31)	217 (48.65)	246 (50.20)	2333 (44.98)
Non-Hispanic Black	299 (12.81)	123 (18.87)	103 (23.79)	173 (20.79)	65 (14.57)	98 (20.00)	861 (16.60)
Others	1019 (43.66)	230 (35.28)	127 (29.33)	307 (36.90)	164 (36.77)	146 (29.80)	1993 (38.42)
Married, n (%) ^†^	1448 (62.58)	396 (61.59)	223 (52.22)	494 (60.24)	265 (59.95)	226 (46.60)	3052 (59.48)
College degree or higher, n (%) ^‡^	1409 (60.39)	337 (51.77)	225 (51.96)	374 (44.95)	175 (39.59)	184 (37.55)	2704 (52.19)
Current smoker, n (%)	270 (11.57)	177 (27.15)	144 (33.26)	294 (35.34)	137 (30.72)	173 (35.31)	1195 (23.04)
Current drinker, n (%) ^§^	1563 (82.48)	434 (77.36)	291 (78.86)	563 (77.76)	266 (71.31)	256 (64.32)	3373 (78.08)
Physical activity, n (%)							
Sedentary	1007 (43.14)	316 (48.47)	205 (47.34)	456 (54.81)	262 (58.74)	328 (66.94)	2574 (49.62)
Moderate	674 (28.88)	192 (29.45)	141 (32.56)	234 (28.13)	133 (29.82)	109 (22.24)	1483 (28.59)
Vigorous	653 (27.98)	144 (22.09)	87 (20.09)	142 (17.07)	51 (11.43)	53 (10.82)	1130 (21.79)
BMI category, n (%) ^¶^							
Normal	597 (25.74)	154 (23.91)	87 (20.23)	197 (24.02)	119 (26.92)	128 (27.06)	1282 (25.00)
Overweight	852 (36.74)	253 (39.29)	171 (39.77)	349 (42.56)	197 (44.57)	169 (35.73)	1991 (38.83)
Obesity	870 (37.52)	237 (36.80)	172 (40.00)	274 (33.41)	126 (28.51)	176 (37.21)	1855 (36.17)
Waist circumference (cm), mean (SD) ^††^	97.70 (14.60)	100.37 (13.85)	101.54 (14.02)	101.23 (13.26)	101.48 (13.20)	101.55 (15.14)	99.60 (14.29)
Systolic BP (mmHg), mean (SD) ^‡‡^	121.36 (15.19)	129.22 (16.55)	131.05 (17.63)	136.79 (19.66)	150.43 (24.83)	131.13 (22.36)	129.05 (20.05)
Diastolic BP (mmHg), mean (SD) ^§§^	74.69 (10.39)	76.86 (11.67)	76.63 (10.54)	75.81 (12.83)	74.69 (14.92)	73.76 (13.01)	75.22 (11.71)
Hypertension, n (%) ^¶¶^	844 (36.18)	262 (40.18)	187 (43.19)	348 (42.03)	218 (49.32)	303 (62.35)	2162 (41.79)
TC to HDL ratio, mean (SD) ^†††^	4.21 (1.28)	4.74 (1.49)	4.99 (1.87)	4.90 (1.78)	5.08 (2.38)	4.36 (1.48)	4.54 (1.62)
High cholesterol, n (%) ^‡‡‡^	1719 (77.99)	470 (77.30)	315 (78.36)	579 (76.59)	279 (70.45)	292 (66.82)	3654 (76.08)
Diabetes, n (%)	80 (3.43)	19 (2.91)	24 (5.54)	77 (9.25)	98 (21.97)	57 (11.63)	355 (6.84)
Number of concurrent conditions including hypertension, high cholesterol, and diabetes, n (%)							
One	2032 (87.06)	553 (84.82)	346 (79.91)	672 (80.77)	319 (71.52)	341 (69.59)	4263 (82.19)
Two or three	302 (12.94)	99 (15.18)	87 (20.09)	160 (19.23)	127 (28.48)	149 (30.41)	924 (17.81)

^†^: 5131 participants without missing data were included; ^‡^: 5181 participants without missing data were included; ^§^: 4320 participants without missing data were included; ^¶^: 5128 participants without missing data were included; ^††^: 5048 participants without missing data were included; ^‡‡^: 5170 participants without missing data were included; ^§§^: 5158 participants without missing data were included; ^¶¶^: 5174 participants without missing data were included; ^†††^: 5157 participants without missing data were included; ^‡‡‡^: 4803 participants without missing data were included. Abbreviations: SD, standard deviation; BMI, body mass index; TC, total cholesterol; HDL, high-density lipoprotein; BP, blood pressure.

**Table 2:** Distribution of CVD risk across age, gender, and race groups.

	Having Known CVD or a 10-Year Predicted Risk ≥ 7.5%		10-Year Predicted Risk < 7.5%		*p* Value
	Number	Percentage (95% CI)	Number	Percentage (95% CI)	
Age group					<0.001
40 to 49 years	273	13.03 (11.07 to 14.98)	1605	86.97 (85.02 to 88.93)	
50 to 59 years	528	30.28 (27.46 to 33.09)	1036	69.72 (66.91 to 72.54)	
60 to 69 years	836	65.50 (61.35 to 69.64)	338	34.50 (30.36 to 38.65)	
70 to 79 years	564	97.86 (96.16 to 99.56)	7	2.14 (0.44 to 3.84)	
Gender					<0.001
Male	1450	45.13 (42.56 to 47.69)	1203	54.87 (52.31 to 57.44)	
Female	751	24.29 (22.22 to 26.36)	1783	75.71 (73.64 to 77.78)	
Race					<0.001
Non-Hispanic White	1018	35.20 (33.08 to 37.32)	1315	64.80 (62.68 to 66.92)	
Non-Hispanic Black	439	42.15 (38.47 to 45.83)	422	57.85 (54.17 to 61.53)	
Others	744	30.32 (27.37 to 33.27)	1249	69.68 (66.73 to 72.63)	
Total	2201	34.83 (33.15 to 36.51)	2986	65.17 (63.49 to 66.85)	

**Table 3:** Comparison of risk factors in patients with hypertension, high cholesterol, and/or diabetes who were untreated and at high CVD risk vs. those who were treated.

Risk Factors	Untreated and High-Risk (n = 2201)	Treated (n = 14,926)	Weighted OR (95% CI) for Untreated and High-Risk	*p* Value from Multivariable Regression Analysis
Age group, n (%)				
40 to 54 years	517 (23.49)	3573 (23.95)	-	-
55 to 64 years	735 (33.39)	4625 (30.99)	1.35 (1.10 to 1.66)	0.004
≥65 years	949 (43.12)	6726 (45.06)	1.84 (1.51 to 2.24)	<0.001
Male, n (%)	1450 (65.88)	7259 (48.63)	2.12 (1.81 to 2.48)	<0.001
Race, n (%)				
Non-Hispanic Black	439 (19.95)	3967 (26.58)	-	-
Non-Hispanic White	1018 (46.25)	6331 (42.42)	1.27 (1.07 to 1.49)	0.006
Others	744 (33.80)	4628 (31.01)	1.47 (1.21 to 1.79)	<0.001
Unmarried, n (%) ^a^	966 (44.43)	6114 (41.32)	1.50 (1.29 to 1.76)	<0.001
College degree or higher, n (%) ^b^	958 (43.60)	6733 (45.18)	1.03 (0.88 to 1.21)	0.708
Current smoker, n (%) ^c^	748 (33.98)	2501 (16.77)	2.62 (2.24 to 3.06)	<0.001
Current drinker, n (%) ^d^	1376 (73.82)	7432 (66.26)	1.04 (0.88 to 1.24)	0.636
Physical activity, n (%) ^e^				
Sedentary	1251 (56.84)	8634 (57.87)	-	-
Moderate	617 (28.03)	4387 (29.41)	1.01 (0.86 to 1.20)	0.875
Vigorous	333 (15.13)	1898 (12.72)	1.09 (0.85 to 1.40)	0.488
BMI category, n (%) ^f^				
Obesity	748 (34.55)	6997 (49.95)	-	-
Overweight	886 (40.92)	4664 (33.30)	1.28 (1.03 to 1.59)	0.024
Normal	531 (24.53)	2346 (16.75)	1.45 (1.12 to 1.88)	0.005
Waist circumference, n (%)				
≥110 cm	544 (24.72)	4835 (32.39)	-	-
100 to 109 cm	592 (26.90)	3680 (24.65)	1.24 (1.02 to 1.51)	0.033
90 to 99 cm	597 (27.12)	3124 (20.93)	1.50 (1.18 to 1.91)	0.001
<90 cm	468 (21.26)	3287 (22.02)	1.50 (1.11 to 2.03)	0.009
Non-diabetic, n (%) ^g^	1945 (88.37)	9785 (65.60)	3.88 (2.94 to 5.11)	<0.001
TC to HDL ratio, n (%)				
<5.0	1310 (59.52)	12,150 (81.40)	-	-
≥5.0	891 (40.48)	2776 (18.60)	3.74 (3.20 to 4.37)	<0.001
Systolic BP (mmHg), n (%)				
<140 mmHg	1356 (61.61)	10,603 (71.04)	-	-
≥140 mmHg	845 (38.39)	4323 (28.96)	1.53 (1.32 to 1.76)	<0.001

^a^: 16,970 participants without missing data were included; ^b^: 17,098 participants without missing data were included; ^c^: 17,117 participants without missing data were included; ^d^: 13,081 participants without missing data were included; ^e^: 17,120 participants without missing data were included; ^f^: 16,172 participants without missing data were included; ^g^: 17,118 participants without missing data were included. Abbreviations: OR, odds ratio; CI, confidence interval; BMI, body mass index; TC, total cholesterol; HDL, high-density lipoprotein; BP, blood pressure. (The percentages are column percentages).

## Data Availability

The datasets supporting the conclusions of this article are available on the NHANES website at https://wwwn.cdc.gov/nchs/nhanes/default.aspx (accessed on 18 March 2024).

## Supplementary Materials

The following supporting information can be downloaded at: https://www.mdpi.com/article/10.3390/jcdd11090268/s1, Table S1: Number and percentage of individuals above treatment threshold and below treatment threshold across age, gender, and race groups; Table S2: Comparison of risk factors in patients with hypertension, high cholesterol, and/or diabetes who were untreated and at high CVD risk vs. who were treated: sensitivity analysis with missing data imputed. References [8,15,16] are cited in the Supplementary Materials.
