# Supplementary material for: Risk of Cardiovascular Events in Adults Aged 40 to 79 Years with Diagnosed Hypertension, High Cholesterol, and/or Diabetes but Not on Medications: Findings from Nationwide Cross-Sectional Studies
Source: J Cardiovasc Dev Dis. 2024 Aug 29;11(9):268. doi: 10.3390/jcdd11090268 (PMC11432436; doi:10.3390/jcdd11090268)
Supplement: Supplementary file 1 [file jcdd-11-00268-s001.zip › jcdd-3127670-supplementary.pdf]

Table S1. Number and percentage of individuals above treatment threshold and below treatment threshold across age, gender, and race groups

	Above treatment threshold[a]		Below treatment threshold		*P* value
	Number	Percentage (95% CI)	Number	Percentage (95% CI)	
Age group					<0.001
40 to 49 years	280	13.53 (11.55 to 15.50)	1598	86.47 (84.50 to 88.45)	
50 to 59 years	498	28.37 (25.63 to 31.11)	1066	71.63 (68.89 to 74.37)	
60 to 69 years	794	62.28 (58.02 to 66.54)	380	37.72 (33.46 to 41.98)	
70 to 79 years	562	97.13 (95.01 to 99.25)	9	2.87 (0.75 to 4.99)	
Gender					<0.001
Male	1390	43.53 (40.95 to 46.10)	1263	56.47 (53.90 to 59.05)	
Female	744	23.78 (21.84 to 25.72)	1790	76.22 (74.28 to 78.16)	
Race					<0.001
Non-Hispanic White	992	34.21 (32.19 to 36.23)	1341	65.79 (63.77 to 67.81)	
Non-Hispanic Black	414	39.95 (36.45 to 43.45)	447	60.05 (56.55 to 63.55)	
Others	728	29.46 (26.40 to 32.52)	1265	70.54 (67.48 to 73.60)	
Total	2134	33.77 (32.15 to 35.39)	3053	66.23 (64.61 to 67.85)	

[a] According to current guidelines, drug treatment would be recommended to a person if the person fulfills any of the following criteria: 1) having existing major CVDs[1,2]; 2) having concurrent high cholesterol and diabetes or concurrent hypertension and diabetes, regardless of the predicted 10-year CVD risk[3]; 3) without high cholesterol, but had hypertension and a 10-year predicted risk ≥10%[3]; or 4) not fulfilling criteria 1 to 3, but having a 10-year predicted risk ≥7.5%[3].

References

1. Grundy SM, Stone NJ, Bailey AL, et al. 2018 AHA/ACC/AACVPR/AAPA/ABC/ACPM/ADA/AGS/APhA/ASPC/NLA/PCNA Guideline on the Management of Blood Cholesterol: A Report of the American College of Cardiology/American Heart Association Task Force on Clinical Practice Guidelines. *J Am Coll Cardiol*. Jun 25 2019;73(24):e285-e350. doi:10.1016/j.jacc.2018.11.003
2. Whelton PK, Carey RM, Aronow WS, et al. 2017 ACC/AHA/AAPA/ABC/ACPM/AGS/APhA/ASH/ASPC/NMA/PCNA Guideline for the Prevention, Detection, Evaluation, and Management of High Blood Pressure in Adults: A Report of the American College of Cardiology/American Heart Association Task Force on Clinical Practice Guidelines. *J Am Coll Cardiol*. May 15 2018;71(19):e127-e248. doi:10.1016/j.jacc.2017.11.006
3. Arnett DK, Blumenthal RS, Albert MA, et al. 2019 ACC/AHA Guideline on the Primary Prevention of Cardiovascular Disease: A Report of the American College of Cardiology/American Heart Association Task Force on Clinical Practice Guidelines. *Circulation*. Sep 10 2019;140(11):e596-e646. doi:10.1161/CIR.0000000000000678

Table S2. Comparison of risk factors in patients with hypertension, high cholesterol, and/or diabetes who were untreated and at high CVD risk vs. who were treated: sensitivity analysis with missing data imputed

Risk factors	Untreated and High-risk (n=2201)	Treated (n=14926)	Weighted OR (95% CI) for Untreated and High-risk	*P* value from multivariable regression analysis
Age group, n (%)				
40 to 54 years	517 (23.49)	3573 (23.95)	-	-
55 to 64 years	735 (33.39)	4625 (30.99)	1.41 (1.17 to 1.69)	<0.001
≥65 years	949 (43.12)	6726 (45.06)	1.86 (1.56 to 2.23)	<0.001
Male, n (%)	1450 (65.88)	7259 (48.63)	2.02 (1.77 to 2.31)	<0.001
Race, n (%)				
Non-Hispanic Black	439 (19.95)	3967 (26.58)	-	-
Non-Hispanic White	1018 (46.25)	6331 (42.42)	1.23 (1.06 to 1.42)	0.006
Others	744 (33.80)	4628 (31.01)	1.42 (1.20 to 1.69)	<0.001
Unmarried, n (%)	977 (44.39)	6171 (41.34)	1.45 (1.26 to 1.67)	<0.001
College degree or higher, n (%)	960 (43.62)	6740 (45.16)	1.04 (0.90 to 1.20)	0.606
Current smoker, n (%)	748 (33.98)	2501 (16.76)	2.60 (2.25 to 2.99)	<0.001
Current drinker, n (%)	1618 (73.51)	9648 (64.64)	1.12 (0.96 to 1.31)	0.138
Physical activity, n (%)				
Sedentary	1251 (56.84)	8638 (57.87)	-	-
Moderate	617 (28.03)	4389 (29.41)	1.07 (0.92 to 1.24)	0.383
Vigorous	333 (15.13)	1899 (12.72)	1.19 (0.95 to 1.49)	0.139
BMI category, n (%)				
Obesity	756 (34.35)	7074 (47.39)	-	-
Overweight	900 (40.89)	4968 (33.28)	1.42 (1.17 to 1.71)	<0.001
Normal	545 (24.76)	2884 (19.32)	1.64 (1.32 to 2.03)	<0.001
Waist circumference, n (%)				
≥110 cm	544 (24.72)	4835 (32.39)	-	-
100 to 109 cm	592 (26.90)	3680 (24.65)	1.16 (0.96 to 1.39)	0.117
90 to 99 cm	597 (27.12)	3124 (20.93)	1.30 (1.05 to 1.63)	0.019
<90 cm	468 (21.26)	3287 (22.02)	1.22 (0.94 to 1.58)	0.133
Non-diabetic, n (%)	1945 (88.37)	9792 (65.60)	3.71 (2.96 to 4.64)	<0.001
TC to HDL ratio, n (%)				
<5.0	1310 (59.52)	12150 (81.40)	-	-
≥5.0	891 (40.48)	2776 (18.60)	3.57 (3.10 to 4.12)	<0.001
Systolic BP (mmHg), n (%)				
<140 mmHg	1356 (61.61)	10603 (71.04)	-	-
≥140 mmHg	845 (38.39)	4323 (28.96)	1.53 (1.34 to 1.74)	<0.001
